# Changes in RNA secondary structure affect NS1 protein expression during early stage influenza virus infection

**DOI:** 10.1186/s12985-019-1271-0

**Published:** 2019-12-21

**Authors:** Irina Baranovskaya, Mariia Sergeeva, Artem Fadeev, Renata Kadirova, Anna Ivanova, Edward Ramsay, Andrey Vasin

**Affiliations:** 1 Smorodintsev Research Institute of Influenza, 15/17 Prof. Popova Str, Saint Petersburg, 197376 Russia; 2 Peter the Great St. Petersburg Polytechnic University, 29 Polytechnicheskaya Str, Saint Petersburg, 195251 Russia; 3 Global Viral Network, 725 West Lombard St Room S413, Baltimore, MD 21201 USA

**Keywords:** Influenza virus, NS gene, RNA secondary structure, RNA hairpin, NS1 protein, Reverse genetics

## Abstract

RNA secondary structures play a key role in splicing, gene expression, microRNA biogenesis, RNA editing, and other biological processes. The importance of RNA structures has been demonstrated in the life cycle of RNA-containing viruses, including the influenza virus. At least two regions of conserved secondary structure in NS segment (+) RNA are predicted to vary among influenza virus strains with respect to thermodynamic stability; both fall in the NS1 open reading frame. The NS1 protein is involved in multiple virus-host interaction processes, and its main function is to inhibit the cellular immune response to viral infection. Using a reverse genetics approach, four influenza virus strains were constructed featuring mutations that have different effects on RNA secondary structure. Growth curve experiments and ELISA data show that, at least in the first viral replication cycle, mutations G123A and A132G affecting RNA structure in the (82–148) NS RNA region influence NS1 protein expression.

## Introduction

The influenza A virus (IAV) poses a serious threat to human health. Despite significant progress in surveillance and control measures, including the development of antiviral drugs, vaccines and diagnostics, IAV continues to evolve and cause epidemics. IAV belongs to the *Ortomyxoviridae* family, and its genome consists of eight segments of negative sense RNA which encode more than 17 proteins [1]. The smallest segment (NS) encodes two proteins (NS1, NEP), and the switch between corresponding ORFs is regulated by mRNA splicing. NEP is a multi-functional nuclear export protein implicated in mediating the export of vRNPs from the host cell nucleus [2]. NS1 is a protein involved in multiple virus-host interactions [3], and a key function is to antagonize the cell’s interferon system, thereby preventing an innate immune response [4]. NS1 is actively expressed early in infection [5], and genetically-engineered deletions in the NS1 open reading frame result in virus attenuation [6]. The level of NS1 expression in infected cells varies between IAV strains. The virus from the 1918 Spanish influenza pandemic, A/Brevig Mission/1/1918 (H1N1), is characterized by a high level of NS1 protein expression, while other strains often feature more moderate NS1 expression [7]. In addition, A/Brevig Mission/1/1918 (H1N1) NS mRNAs are less efficiently spliced in comparison to other influenza viruses; this may be associated with higher NS1 protein production [8]. The formation of stable secondary structures in NS (+) RNA has been demonstrated earlier by several research groups [9–11]. Two regions, corresponding to RNA positions 82–148 and 497–564, are located near splice sites and form stable RNA secondary structures. It has been predicted that the type of these structures can vary between influenza A virus strains [12]. The first region (82–148) forms multi-branch or stem-loop structures [9, 13], while the second region (497–564) has a tendency to fold into pseudoknot or stem-loop structures [14]. It has been noted that highly virulent avian H5N1 influenza viruses which appeared after the 2005 outbreak carry stable, energetically-favored, hairpin structures in the second region [15]. Other researchers have shown that mutant influenza A viruses with altered RNA structure in the second region display changes in NS mRNA splicing and viral replication in cell culture [16]. In this research, we analyzed the roles in NS1 protein expression and viral reproduction in vitro of mutations that influence conserved RNA secondary structures located in two NS regions.

## Material and methods

### RNA secondary structure analysis

Influenza virus NS gene nucleotide sequences were downloaded from the NCBI Influenza Virus Resource [17] and Influenza Research Database (IRD) [18]. RNA secondary structures were predicted using the RNAfold online tool [19], which does not include pseudoknot prediction.

### Site-directed mutagenesis

The pHW-PR8-NS plasmid, encoding the NS segment of the A/PR/8/34 (H1N1) virus, was used as a starting point. PCR with primers featuring incompletely overlapping sequences was used to introduce mutations, as described by Zheng et al. [20]. The primers used for PCR site-directed mutagenesis are listed (Additional file 1: Table S1). Mutations were first introduced into the 82–148 region, then into the 497–564 region. Four plasmid variants were obtained, and modifications were verified by Sanger DNA sequencing [21].

### In vitro transcription

RNA fragments of NS regions 82–148 and 496–564 were prepared by in vitro transcription. At first, PCR products containing the regions of interest were amplified from modified pHW-PR8-NS plasmids, and T7 promoter sequences were added via the primers used (Additional file 2: Table S2). Next, RNA regions were synthesized by T7 RNA Polymerase (Promega, #P207B) according to the enzyme manufacturer’s instructions. Following RNA synthesis, template DNA was removed by subsequent digestion with DNase (Promega, #M6101). Transcripts were purified by double isopropanol precipitation [22].

### Electrophoretic mobility under native and denaturing conditions

For analysis under denaturing conditions, 1 μg of each RNA sample was mixed with an equal volume of 2x RNA Gel Loading Dye (Thermo Scientific, #R0641), incubated for 30 min at room temperature, and then heated for 3 min at 95 °C. Samples were subsequently loaded onto a PAGE (12.5% polyacrylamide/8 M urea) and run in TBE buffer at 55 °C. For analysis under native conditions, samples were incubated for 2 min at 90 °C, followed by slow cooling (approx. 2 degrees per 20 s) until 37 °C, and further incubated for 25 min at 37 °C, as described by the Moss group [9]. Samples were then mixed with 6x gel loading buffer without formamide (0.25% bromophenol blue (w/v), 0.25% xylene cyanol FF (w/v), 40% sucrose (w/v)), loaded onto a 12.5% polyacrylamide gel, and run in 0.5xTBE buffer at 37 °C. Gels were stained with silver nitrate [23], and images were captured using a ChemiDoc XRS+ System (Bio-Rad).

### Cell culture

MDCK London line cells (International Reagent Resource, #FR-58) were cultivated in Alpha MEM (Biolot) supplemented with 2 mM L-glutamine and 10% “SC-biol” FBS (Biolot). A549 cells were cultivated in DMEM/F12 (Gibco) media supplemented with 2% GlutaMAX (Gibco) and 10% FBS (Gibco). Vero cells (ATCC, #CCL-81) were adapted to serum-free medium and cultivated in OptiPro SFM (Gibco) supplemented with 2% GlutaMAX (Gibco).

### Generation of viruses

Viruses were obtained by reverse genetics [24]. Plasmids encoding 8 gene segments of A/PR/8/34 (H1N1) including mutated NS segments were transfected into Vero cells by Nucleofector technology (Amaxa #VCA-1003). Viruses were collected from the supernatant 72 h post transfection, and working stock was generated by one passage in Vero cells at moi = 0.01. Virus infectious activity was measured by titration in Vero cells. The 50% Tissue Culture Infectious Dose (TCID_50_) endpoint was calculated by the Reed and Muench method [25]. Virus genome sequences, including the mutations introduced, were confirmed by next generation sequencing (NGS).Full-genome amplification was performed according to Zhou et al. [26]). Nextera XT (Illumina) sample preparation was used to obtain libraries for NGS; full-length genome sequences were obtained using Illumina MiSeq.

### Viral growth kinetics

Multi-cycle viral growth kinetics were measured in Vero, MDCK, and A549 cells (overnight confluent, 6-well plate format, triplicates) by infection with virus at moi = 0.001 (TCID_50_/cell). After 1 h of virus adsorption at room temperature, medium was replaced. Serum free culture media (see above) was supplemented with 1% antibiotic-antimycotic (Gibco) and TPCK-trypsin (Sigma) at: 2.5 μg/ml for MDCK; and 0.5 μg/ml for A549 and Vero. Viral progeny was collected from the supernatant at the indicated time points, and infectious titers were measured.

### Elisa

MDCK or A549 monolayers (overnight confluent, 48-well plate format, triplicates) were infected with assembled viruses at moi = 10. After virus adsorption at 37 °C, the inoculum was removed and replaced with serum free medium containing antibiotic-antimycotic and TPCK-trypsin. Infected cells were incubated at 37 °C for the indicated time periods and then fixed with 80% acetone in DPBS (ice cold at application) for 15 min at room temperature. Fixed cells were washed three times with phosphate buffered saline with 0.1% tween-20 (PBST) and blocked with 5% milk in PBST for 6 h at 4 °C. After 2 washes, plates were incubated at 4 °C overnight with mouse polyclonal serum raised against recombinant NS1_(1–124)_ protein. Plates were then washed three times with PBST, and goat anti-mouse secondary antibody (Bio-Rad) was added (1 μg/ml final), followed by incubation at 37 °C for 1 h. Immunoreactivities were analyzed by adding TMB Peroxidase EIA Substrate (Bio-Rad), and reactions were stopped with 2 N H_2_SO_4_. Optical density was measured at 450 nm with removal of noise (measured at 655 nm) using a CLARIOstar multi-function reader (BMG Labtech).

### Real-time PCR analysis

MDCK or A549 cell monolayers (overnight confluent, 6-well plate format, triplicates) were used for infection at moi = 1. After virus adsorption at 37 °C, the inoculum was removed and replaced with serum free medium. At the indicated time points, media was removed, and total RNAs were extracted from cells using the RNeasy Mini kit (Qiagen) according to the manufacturer’s protocol. Complementary DNA was synthesized from 500 ng of total RNA using M-MLV Reverse Transcriptase (Promega) and oligo(dT_16_) primers (DNA synthesis, Russia) according to the manufacturer’s protocol. Quantitative real-time PCR analysis was performed using (2x) BioMaster HS-qPCR SYBR Blue (Biolabmix). The primers used are provided (Additional file 3: Table S3). NS1 gene expression was calculated by the 2^−ΔΔCt^method, and GAPDH mRNA was used for normalization. The average value of the “reference sample” replicates (“1–1” sample at 1 h post-infection, h.p.i.) was designated as expression level ‘1.0’ and used for relative expression quantitation.

### Statistics

Statistical analysis was performed using GraphPad Prizm 6.01 software. One-way ANOVA was performed to evaluate differences in virus infectious activity. Two-way ANOVA with Tukey post test was used to evaluate the significance of any differences between viruses at specific time points, revealed by ELISA and RT-PCR. A value of 0.05 was used as the threshold of significance. All samples were processed in triplicate, and presented on graphs by mean ± sd.

## Results and discussion

**Fig. 1:**
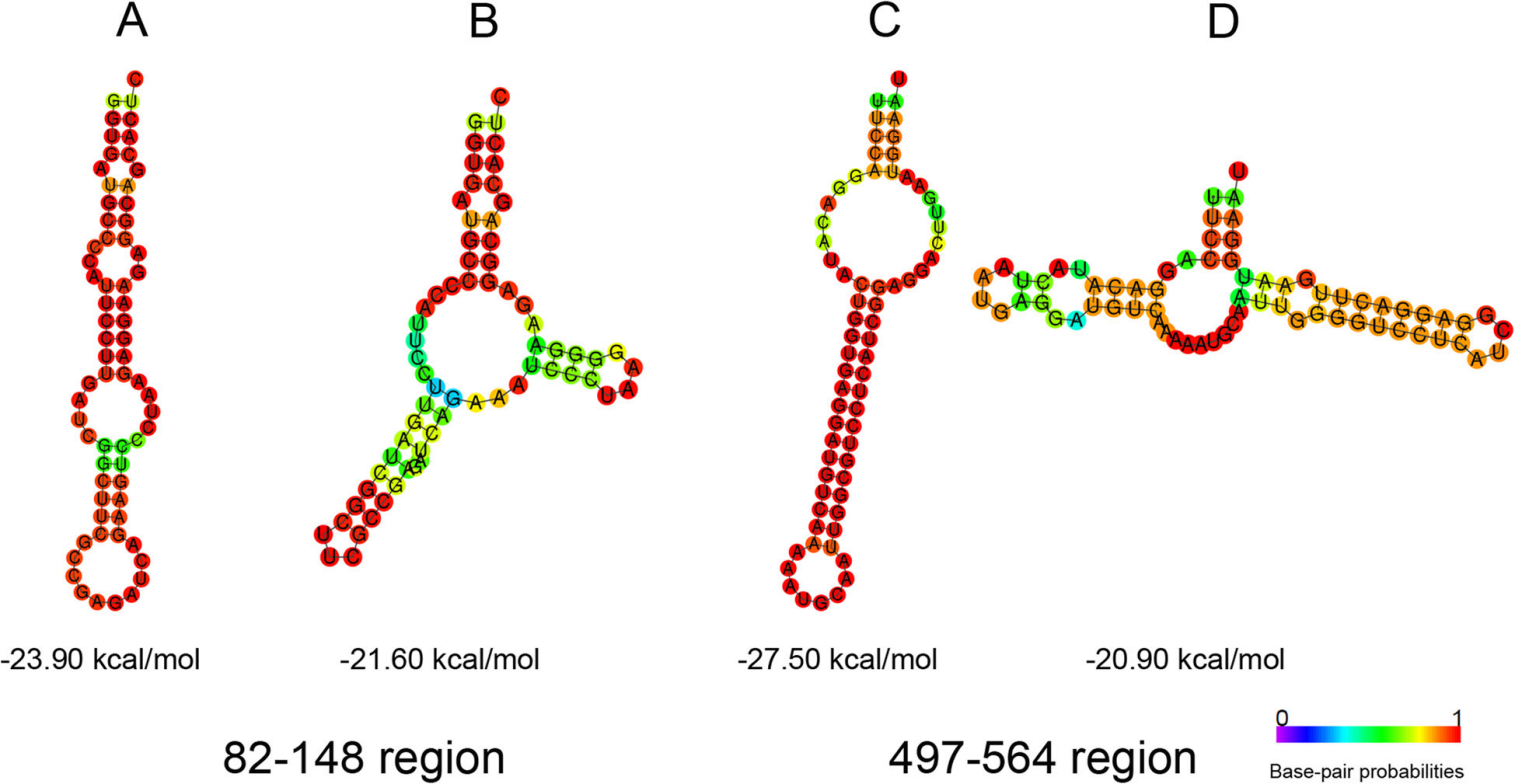
Predicted secondary structures of NS gene segment RNA regions 82–148 and 497–564 without and with mutations (**a**) 82–148 original sequence of A/Brevig Mission/1/1918 (H1N1) (hairpin), (**b**) 82–148 mutated sequence featuring G123A and A132G substitutions, **(c)**497–564 original sequence of A/Vietnam/1194/2004 (H5N1) (hairpin), **(d)** 497–564 mutated sequence featuring G511A, G512A and C537G substitutions

RNA secondary structures at NS nucleotide regions 82–148 and 497–564 have been previously predicted for a large number of influenza A viruses [12]. In order to investigate whether these secondary structures are indeed involved in viral replication or NS1 protein production, we chose sequences from A/Brevig Mission/1/1918 (H1N1)and A/Vietnam/1194/2004 (H5N1) pathogenic influenza virus strains characterized by NS (+) RNA hairpin structure in the 82–148 and 497–564 regions, respectively. Based on our previous RNA structure analysis of human influenza A viruses [12], we chose mutations that change hairpin structures in both regions (Fig. 1). In the 82–148 region we introduced two synonymous mutations: G123A and A132G. In order to change hairpin structure in the 497–564 NS (+) RNA region, we introduced three nucleotide substitutions(G511A, G512A and C537G) that lead to one amino acid mutation in the NS1 open reading frame (G166 N) and two mutations in the NEP ORF (V14 M and A22G) (Additional file 4: Figure S1).

In order to obtain four influenza virus strains that would differ only by NS RNA secondary structures in the regions of interest, we used the well characterized influenza virus A/PR/8/34 (H1N1) laboratory strain as the backbone and performed site-directed mutagenesis on the NS gene. Mutations were introduced into a plasmid encoding the A/PR/8/34 (H1N1) NS gene to obtain sequences at regions 82–148 and 497–564 identical to those of the selected sequences. We obtained 4 plasmids encoding the A/PR/8/34 virus NS with different RNA secondary structures in regions 82–148 (region 1) and 497–564 (region 2). The naming of the 4 plasmids, according to the structures present, is as follows: “1–1” (hairpin in both regions); “1–0” (hairpin at region 1); “0–1” (hairpin at region 2); and “0–0” (without hairpins).

**Fig. 2:**
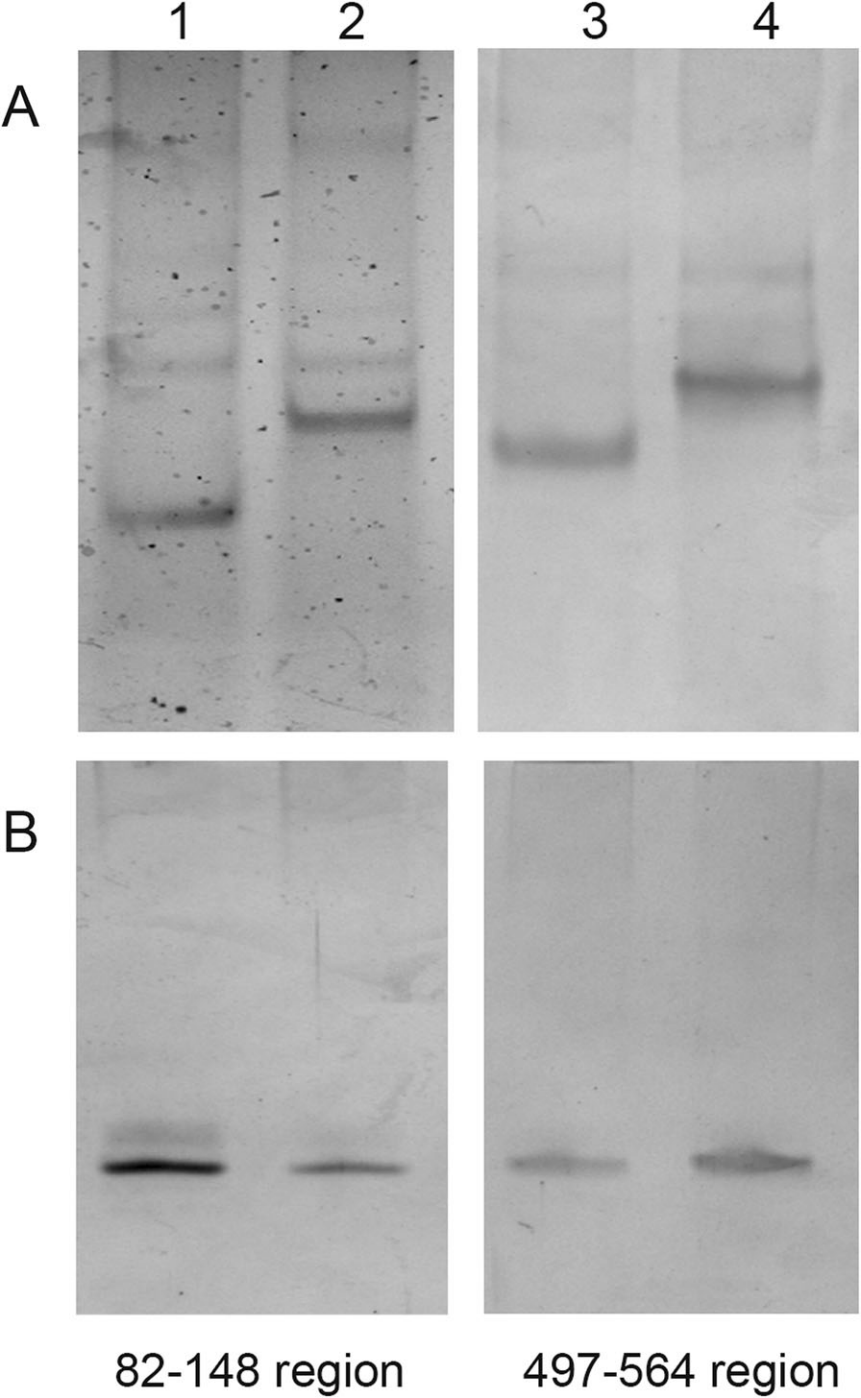
Correlation between electrophoretic mobility of NS viral RNA fragments and predicted secondary structures. PAGE of RNA fragments under native conditions (**a**) and denaturing conditions (**b**). Lane 1–82-148 original sequence, lane 2–82-148 mutated sequence, lane 3–497-564 original sequence, lane 4–497-564 mutated sequence

To confirm the presence of the predicted RNA secondary structure differences, we performed in vitro transcription of the regions of interest, followed by electrophoresis. Native electrophoresis provides an approach for identification of RNA structure differences in samples and has been used for this purpose in previous publications [9, 15]. We observed that the RNA fragments had identical mobilities under denaturing conditions, yet different mobilities under native conditions (Fig. 2). As expected, sequences predicted to fold in more compact hairpin structures moved faster under native conditions. Thus, we confirmed the existence of RNA secondary structure differences in the NS RNA fragments 82–148 and 497–564 obtained from the constructed NS gene plasmids.

**Fig. 3:**
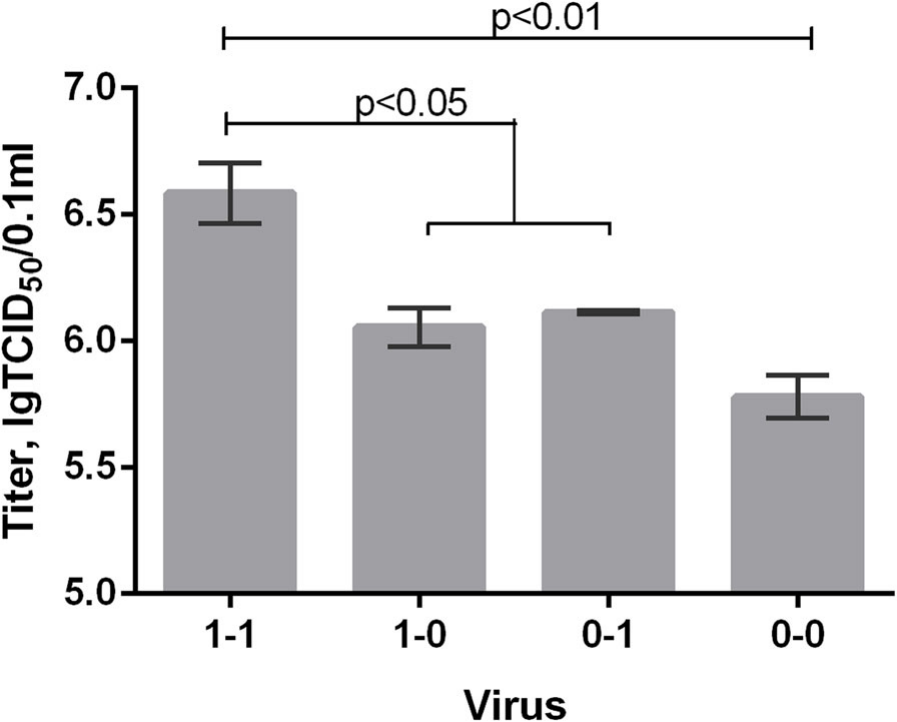
Infectious activity of assembled viruses featuring different NS RNA structures after their rescue by the reverse genetics technique Viruses are: “1–1” - hairpin in regions 1 and 2; “1–0” - hairpin at region 1; “0–1” - hairpin at region 2; and “0–0” - without hairpins. Assembled virus titers were determined 72 h post transfection in Vero cell culture. Error bars represent mean ± s.d (*n* = 3 in two independent experiments). Differences were analyzed by one-way ANOVA with Tukey’s Multiple Comparison Test

We next determined whether these RNA structure differences are involved in viral replication. We rescued 4 influenza viruses that have predicted combinations of NS gene RNA secondary structures. Using the four constructed NS gene plasmids and seven other plasmids encoding the remaining A/PR/8/34 (H1N1) genes, we transfected Vero cells to obtain viruses by reverse genetics. Three days post cell transfection, we collected culture supernatants containing assembled viruses and determined their infectious activity by titration in Vero cells. Virus “1–1”, containing hairpin structures in both NS regions, had higher titers than other viruses (Fig. 3). In addition, the lowest infectious titer corresponded to virus “0–0”, which doesn’t carry hairpin NS RNA secondary structures at either region.

**Fig. 4:**
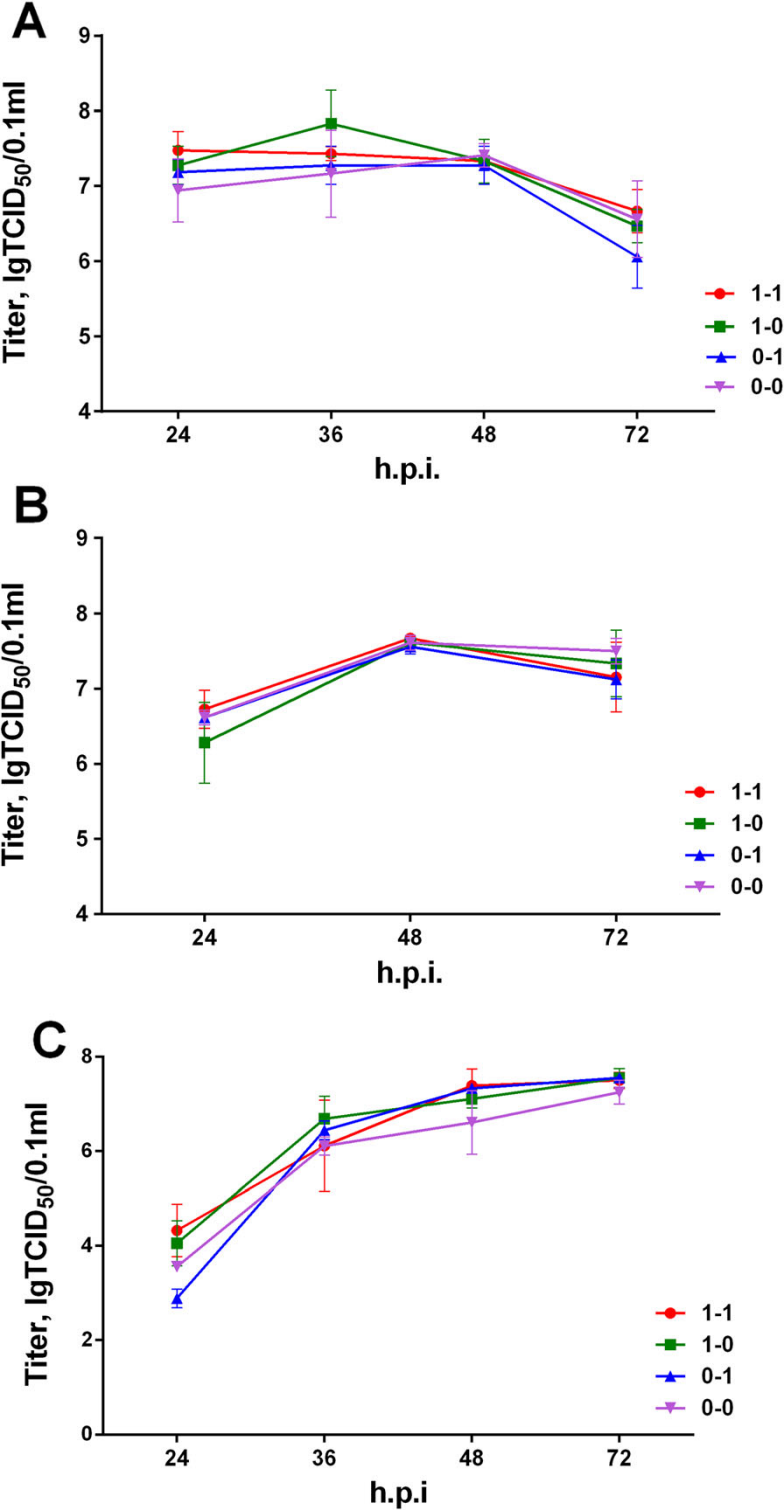
Multi-cycle growth curves of assembled viruses featuring different NS RNA structures. MDCK (**a**), Vero (**b**)**,** and A549 **(c)** cells were infected at moi = 0.001 TCID_50_/cell. The infectious activity of virus progeny was measured at the indicated time points by TCID_50_ assay. Error bars represent mean ± SD (*n* = 3)

Further, we evaluated the replication of the assembled viruses using multi-cycle growth curves in different cell cultures. Vero, MDCK, or A549 cells were infected at low multiplicity of infection (moi = 0.001), and the titers of viral progeny (from infected cell supernatants) were determined at 24, 36, 48, and 72 h post infection (h.p.i). No significant differences were identified between the viruses as a function of NS gene RNA structures (Fig. 4). The only difference seen was between host cell types; infection in A549 cells developed more slowly, as indicated by the lower TCID_50_ values for the first time point. Despite the observed uniformity of multi-cycle infection, the fact that viruses with various NS RNA structures differed from each other by infectious activity after transfection, led us to theorize that RNA structure may be important in the early stages of infection. Therefore, we continued our research, yet limiting ourselves to the first infection cycle.

**Fig. 5:**
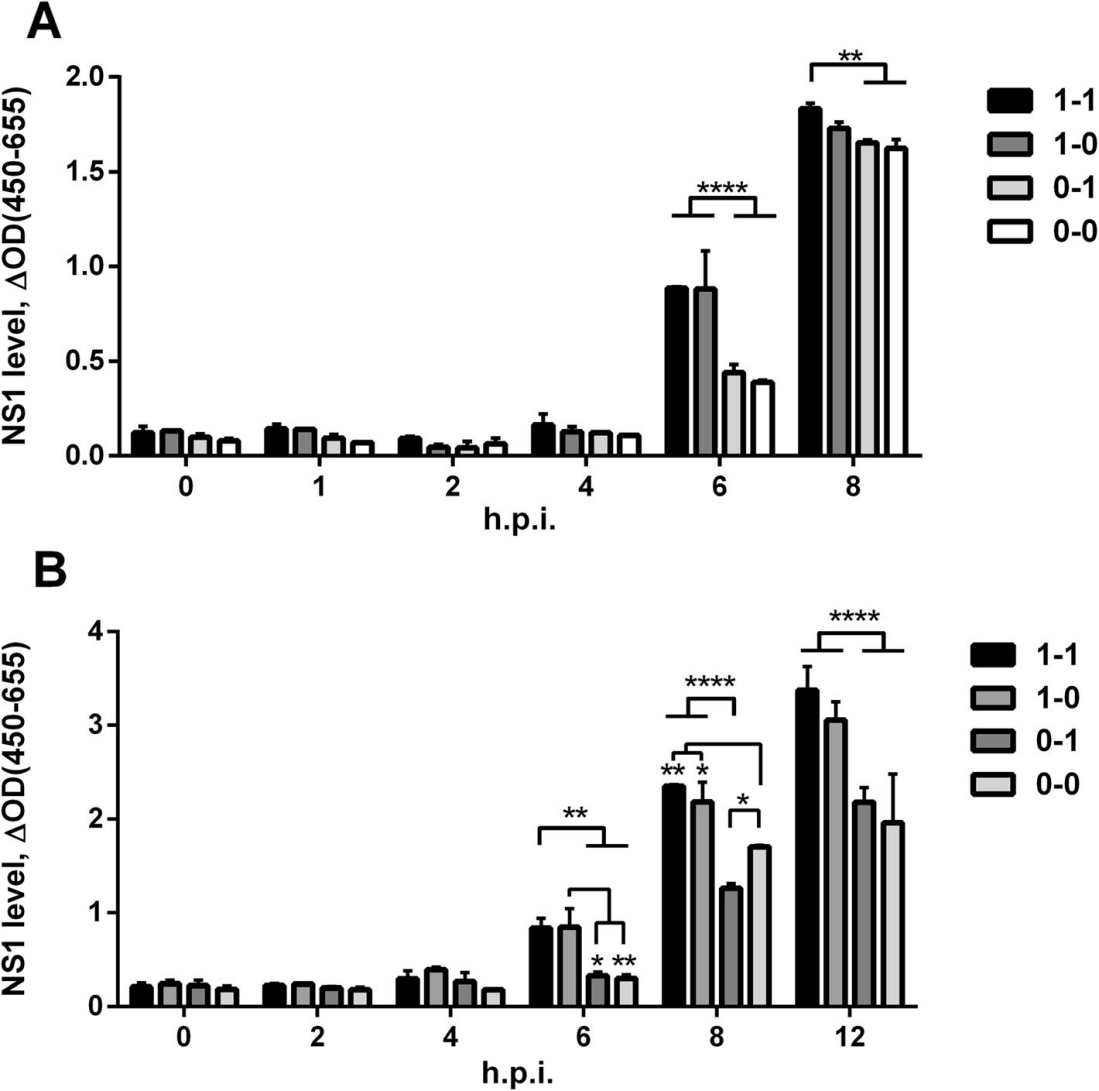
NS1 expression in cells infected by assembled viruses featuring different NS RNA structures. MDCK (**a**) or A549 (**b**)cell cultures were infected with virus at moi = 10 TCID_50_/cell. Infected cells were fixed at the indicated time points, and NS1 protein levels were analyzed by ELISA. Error bars represent mean ± SD. Differences were analyzed by two-way ANOVA with Tukey’s multiple comparisons test (**p < 0.001, ****p < 0.00001)

In order to investigate whether RNA structure affects NS1 expression, we studied NS1 protein accumulation in cells during the first 8–12 h.p.i. Cell cultures were infected at high dose (moi = 10), and cell samples were fixed every 2 h for evaluation of NS1 expression by ELISA (Fig. 5). We observed that NS1 levels were significantly higher at 6 h.p.i. for viruses 1–1 and 1–0, both of which feature a hairpin-type secondary structure in the first NS region. The reduced NS1 protein expression/accumulation observed is likely due to the role of the structures designed (via mutations G123A and A132G in the 82–148 NS RNA region). We cannot, however, definitively rule out that the primary changes (DNA/RNA substitutions) may play some kind of role. The type of structure in the second region had no obvious effect. Observations were similar in MDCK cells and A549 cells.

**Fig. 6:**
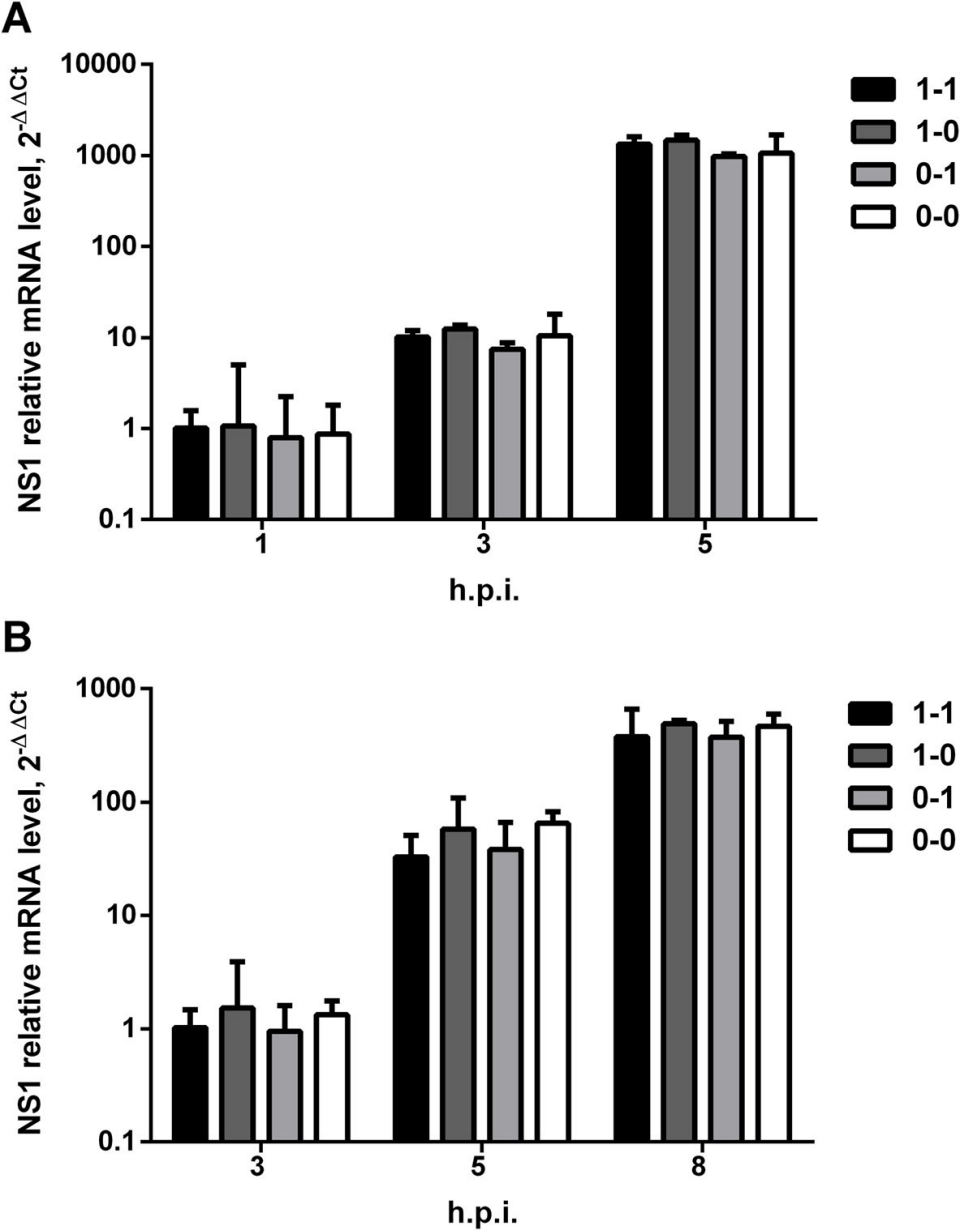
Relative NS1 mRNA expression levels, by RT-qPCR, in cells infected by assembled viruses featuring different NS RNA structures MDCK (**a**) or A549 (**b**) cell cultures were infected at moi = 1 TCID_50_/cell, and NS1 mRNA was measured at the indicated time points. NS1 expression was normalized to GAPDH and presented relative to the “reference sample” (virus “1–1” at the first time point). Error bars represent geometric mean ± 95% CI

We also determined NS1 mRNA levels in cells during the first hours of infection by quantitative RT-PCR. Cells were infected at moi = 1 TCID_50_/cell, and total RNA was extracted at 1–5 h.p.i. for MDCK and 3–8 h.p.i. for A549 cells. NS1 mRNA accumulated more rapidly for MDCK cells than for A549 cells (100 vs 30-fold difference from 3 h.p.i. to 5 h.p.i.) (Fig. 6). Although small differences were observed between viruses at some time points, they did not exceed 2-fold; as such, they were interpreted as biologically insignificant. To address the possibility that NS1 protein expression may also be connected to changes in NS mRNA splicing efficiency, we checked the NEP/NS1 mRNA ratio. The data obtained agreed with work previously reported by Huang et al. [27] and showed that NEP mRNA levels ranged from 20 to 40% of the NS1 mRNA level, with no significant differences seen between viruses (Additional file 5: Figure S2). Thus, we did not observe changes in splicing that should significantly affect NS1 mRNA level or NS1 protein expression.

Due to the fact that significant NS1 expression differences were seen by ELISA, but not by PCR, we assume that the observed effect occurs at the level of translation, not transcription. This result fits with previously published research wherein disruption of the 82–148 hairpin resulted in decreased NS1 protein expression in plasmid transfection experiments [11]. Here, we confirm that the effect also takes place in vitro during viral infection. Our data also supports the suggestion that the RNA hairpin of the A/Brevig Mission/1/1918 virus (hairpin in first region) may promote the production of higher NS1 protein levels, potentially determining the pathogenic properties of that strain. Thus, we consider the hairpin RNA structure in the first NS mRNA region as a potential upregulator of NS1 expression.

Although we sought from the outset to clarify the effects (joint or individual) of both conserved hairpins in NS RNA, the data do not reveal any obvious influences attributable to structure in the second region. Our interest in the effects of secondary structure in the second region (497–564) was especially provoked by earlier work showing that highly virulent H5N1 avian strains tend to possess a stable stem-loop structure in the region [27]. In addition, alterations of RNA structures in the second region have been shown to lead to changes in NS mRNA splicing and attenuated viral replication in cell culture [16]. Therefore, secondary structure at the second region may be involved in processes distinct from regulation of NS1 protein expression, yet still contributing to viral pathogenesis. Thus, a joint influence, arising from both regions of NS RNA secondary structure, should not be ruled out. Further study is needed to precisely determine the individual and joint roles of these structures.

## Supplementary information

**Additional file 1: Table S1.** Primers used in site-directed mutagenesis.

**Additional file 2: Table S2.** Primers used for in vitro transcription.

**Additional file 3: Table S3.** Primers used in qRT-PCR.

**Additional file 4: Figure S1.** NS gene nucleotide alignment of the selected influenza virus strains.

**Additional file 5: Figure S2.** Relative NEP/NS1 mRNA expression levels in cells infected by assembled viruses featuring different NS RNA secondary structures.

## Data Availability

Data and materials are available from the corresponding author on reasonable request.

## Abbreviations

FBS: Fetal Bovine Serum
h.p.i: Hours post infection
IAV: Influenza A virus
IRD: Influenza Research Database
moi: Multiplicity of infection
NGS: Next generation sequencing
PBST: PBS containing 0.05% Tween-20
TCID_50_: 50% Tissue Culture Infectious Dose

## References

[1] Vasin AV, Temkina OA, Egorov VV, Klotchenko SA, Plotnikova MA, Kiselev OI (2014). Molecular mechanisms enhancing the proteome of influenza a viruses: an overview of recently discovered proteins. Virus Res.

[2] Paterson D, Fodor E (2012). Emerging roles for the influenza a virus nuclear export protein (NEP). PLoS Pathog.

[3] Hale BG, Randall RE, Ortín J, Jackson D (2008). The multifunctional NS1 protein of influenza a viruses. J Gen Virol.

[4] García-Sastre A, Egorov A, Matassov D, Brandt S, Levy DE, Durbin JE (1998). Influenza a virus lacking the NS1 gene replicates in interferon-deficient systems. Virology..

[5] Young JF, Desselberger U, Palese P, Ferguson B, Shatzman AR, Rosenberg M (1983). Efficient expression of influenza virus NS1 nonstructural proteins in Escherichia coli. Proc Natl Acad Sci U S A.

[6] Egorov A, Brandt S, Sereinig S, Romanova J, Ferko B, Katinger D (1998). Transfectant influenza a viruses with long deletions in the NS1 protein grow efficiently in Vero cells. J Virol.

[7] Petersen H, Mostafa A, Tantawy MA, Iqbal AA, Hoffmann D, Tallam A (2018). NS segment of a 1918 influenza a virus-descendent enhances replication of H1N1pdm09 and virus-induced cellular immune response in mammalian and avian systems. Front Microbiol.

[8] Backström Winquist E, Abdurahman S, Tranell A, Lindström S, Tingsborg S, Schwartz S (2012). Inefficient splicing of segment 7 and 8 mRNAs is an inherent property of influenza virus a/Brevig Mission/1918/1 (H1N1) that causes elevated expression of NS1 protein. Virology..

[9] Moss WN, Priore SF, Turner DH (2011). Identification of potential conserved RNA secondary structure throughout influenza a coding regions. RNA..

[10] Chursov A, Kopetzky SJ, Leshchiner I, Kondofersky I, Theis FJ, Frishman D (2012). Specific temperature-induced perturbations of secondary mRNA structures are associated with the cold-adapted temperature-sensitive phenotype of influenza a virus. RNA Biol.

[11] Ilyinskii PO, Schmidt T, Lukashev D, Meriin AB, Thoidis G, Frishman D (2009). Importance of mRNA secondary structural elements for the expression of influenza virus genes. Omics.

[12] Vasin AV, Petrova AV, Egorov VV, Plotnikova MA, Klotchenko SA, Karpenko MN (2016). The influenza a virus NS genome segment displays lineage-specific patterns in predicted RNA secondary structure. BMC Res Notes.

[13] Priore SF, Kierzek E, Kierzek R, Baman JR, Moss WN, Dela-Moss LI (2013). Secondary structure of a conserved domain in the intron of influenza a NS1 mRNA. PLoS One.

[14] Gultyaev AP, Olsthoorn RCL (2010). A family of non-classical pseudoknots in influenza a and B viruses. RNA Biol.

[15] Gultyaev AP, Heus HA, Olsthoorn RCL (2007). An RNA conformational shift in recent H5N1 influenza a viruses. Bioinformatics..

[16] Jiang T, Nogales A, Baker SF, Martinez-Sobrido L, Turner DH (2016). Mutations designed by ensemble defect to Misfold conserved RNA structures of influenza a segments 7 and 8 affect splicing and attenuate viral replication in cell culture. PLoS One.

[17] Bao Y, Bolotov P, Dernovoy D, Kiryutin B, Zaslavsky L, Tatusova T (2008). The influenza virus resource at the National Center for biotechnology information. J Virol.

[18] Squires RB, Noronha J, Hunt V, García-Sastre A, Macken C, Baumgarth N (2012). Influenza research database: an integrated bioinformatics resource for influenza research and surveillance. Influenza Other Respir Viruses.

[19] Gruber A. R., Lorenz R., Bernhart S. H., Neubock R., Hofacker I. L. (2008). The Vienna RNA Websuite. Nucleic Acids Research.

[20] Zheng L, Baumann U, Reymond JL (2004). An efficient one-step site-directed and site-saturation mutagenesis protocol. Nucleic Acids Res.

[21] Sanger F, Nicklen S, Coulson AR (1977). DNA sequencing with chain-terminating inhibitors. Proc Natl Acad Sci U S A.

[22] Olsthoorn RCL, Mertens S, Brederode FT, Bol JF (1999). A conformational switch at the 3′ end of a plant virus RNA regulates viral replication. EMBO J.

[23] Blum H, Beier H, Gross HJ (1987). Improved silver staining of plant proteins, RNA and DNA in polyacrylamide gels. Electrophoresis.

[24] Hoffmann E, Neumann G, Kawaoka Y, Hobom G, Webster RG (2000). A DNA transfection system for generation of influenza a virus from eight plasmids. Proc Natl Acad Sci U S A.

[25] Reed LJ, Muench H (1938). A simple method of estimating fifty percent endpoints. Am J Epidemiol.

[26] Zhou B, Donnelly ME, Scholes DT, St George K, Hatta M, Kawaoka Y, Wentworth DE (2009). Single-reaction genomic amplification accelerates sequencing and vaccine production for classical and swine origin human influenza a viruses. J Virol.

[27] Huang X, Zheng M, Wang P, Mok BW, Liu S, Lau SY (2017). An NS-segment exonic splicing enhancer regulates influenza A virus replication in mammalian cells. Nat Commun.

