# Supplementary material for: Changes in RNA secondary structure affect NS1 protein expression during early stage influenza virus infection
Source: Virol J. 2019 Dec 21;16:162. doi: 10.1186/s12985-019-1271-0 (PMC6925897; doi:10.1186/s12985-019-1271-0)
Supplement: Supplementary file 1 — Additional file 1: Table S1. Primers used in site-directed mutagenesis. [file 12985_2019_1271_MOESM1_ESM.docx]

**Table S1.** Primers used in site-directed mutagenesis

| Primer name | Sequence (5'-3') |
| --- | --- |
| F-82-148 (0)_sdm | ggAAgAggCAgCACTCTCggTCTggACATCgAGACAgC |
| R-82-148 (0)_ sdm | gAgAgTgCTgCCTCTTCCCCTTAgggATTTCTgATCTCggCgAAgC |
| F-82-148 (1)_sdm | CTAAgAggAAgAggCAgCACTCTCggTCTggACATCgAg |
| R-82-148 (1)_sdm | gCTgCCTCTTCCTCTTAgggACTTCTgATCTCggCgAAgC |
| F-497-564 (0)_sdm | AATgCAATTggggTCCTCATCggAggACTTgAATggAATgATAAC |
| R-497-564 (0)_sdm | gAggACCCCAATTgCATTTTTgACATCCTCATTAgTATgTCCTgg |
| F-497-564 (1)_sdm | TgCAATTggCgTCCTCATCggAggACTTgAATggAATg |
| R-497-564 (1)_sdm | gAggACgCCAATTgCATTTTTgACATCCTCACCAgTATgTCCTgg |

Introduced mutations are highlighted with green.
