# Supplementary material for: Changes in RNA secondary structure affect NS1 protein expression during early stage influenza virus infection
Source: Virol J. 2019 Dec 21;16:162. doi: 10.1186/s12985-019-1271-0 (PMC6925897; doi:10.1186/s12985-019-1271-0)
Supplement: Supplementary file 3 — Additional file 3: Table S3. Primers used in qRT-PCR. [file 12985_2019_1271_MOESM3_ESM.docx]

**Table S3.** Primers used in qRT-PCR

| Target | Primer name | Primer sequence (5'-3') |
| --- | --- | --- |
| GAPDH  [Jiang et al. 2016] | F-qC_GAPDH(MDCK) | AATGTATCAGTTGTGGATCT |
|  | R-qC_GAPDH(MDCK) | TGCTTCACTACCTTCTTG |
|  | F-qH_GAPDH(A549) | CTCATGACCACAGTCCATGC |
|  | R-qH_GAPDH(A549) | TTCAGCTCAGGGATGACCTT |
| NS1 | F-qNS1 | TACCTAACTGACATGACTCTTGAG |
|  | R-qNS1 | TCGCCTGGTCCATTCTGATAC |

Primers were synthesized by Evrogen (Russia).
