# Supplementary material for: Changes in RNA secondary structure affect NS1 protein expression during early stage influenza virus infection
Source: Virol J. 2019 Dec 21;16:162. doi: 10.1186/s12985-019-1271-0 (PMC6925897; doi:10.1186/s12985-019-1271-0)
Supplement: Supplementary file 4 — Additional file 4: Figure S1. NS gene nucleotide alignment of the selected influenza virus strains. [file 12985_2019_1271_MOESM4_ESM.docx]

**A**

82 148

A/Puerto Rico/8/1934 (H1N1) GGTGATGCCCCATTCCTTGATCGGCTTCGCCGAGATCAGAAATCCCTAAGAGGAAGGGGCAGCACCC

A/Brevig Mission/1/1918(H1N1)GGTGATGCCCCATTCCTTGATCGGCTTCGCCGAGATCAGAAGTCCCTAAGAGGAAGAGGCAGCACTC

mutated sequence GGTGATGCCCCATTCCTTGATCGGCTTCGCCGAGATCAGAAATCCCTAAGGGGAAGAGGCAGCACTC

**B**

497 564

A/Puerto Rico/8/1934 (H1N1) TTCCAGGACATACTGCTGAGGATGTCAAAAATGCAGTTGGAGTCCTCATCGGGGGACTTGAATGGAAT

A/Vietnam/1194/2004 (H5N1) TTCCAGGACATACTGGTGAGGATGTCAAAAATGCAATTGGCGTCCTCATCGGAGGACTTGAATGGAAT

mutated sequence TTCCAGGACATACTAATGAGGATGTCAAAAATGCAATTGGGGTCCTCATCGGAGGACTTGAATGGAAT

**Fig. S1.** Nucleotide alignment of NS gene sequences at the 82-148 (a) and 497-564 (b) regions. All mismatching nucleotides in the PR8 strain were mutated to correspond to sequences of the aforementioned viruses.
