# Supplementary material for: Changes in RNA secondary structure affect NS1 protein expression during early stage influenza virus infection
Source: Virol J. 2019 Dec 21;16:162. doi: 10.1186/s12985-019-1271-0 (PMC6925897; doi:10.1186/s12985-019-1271-0)
Supplement: Supplementary file 5 — Additional file 5: Figure S2. Relative NEP/NS1 mRNA expression levels in cells infected by assembled viruses featuring different NS RNA secondary structures. [file 12985_2019_1271_MOESM5_ESM.docx]

**
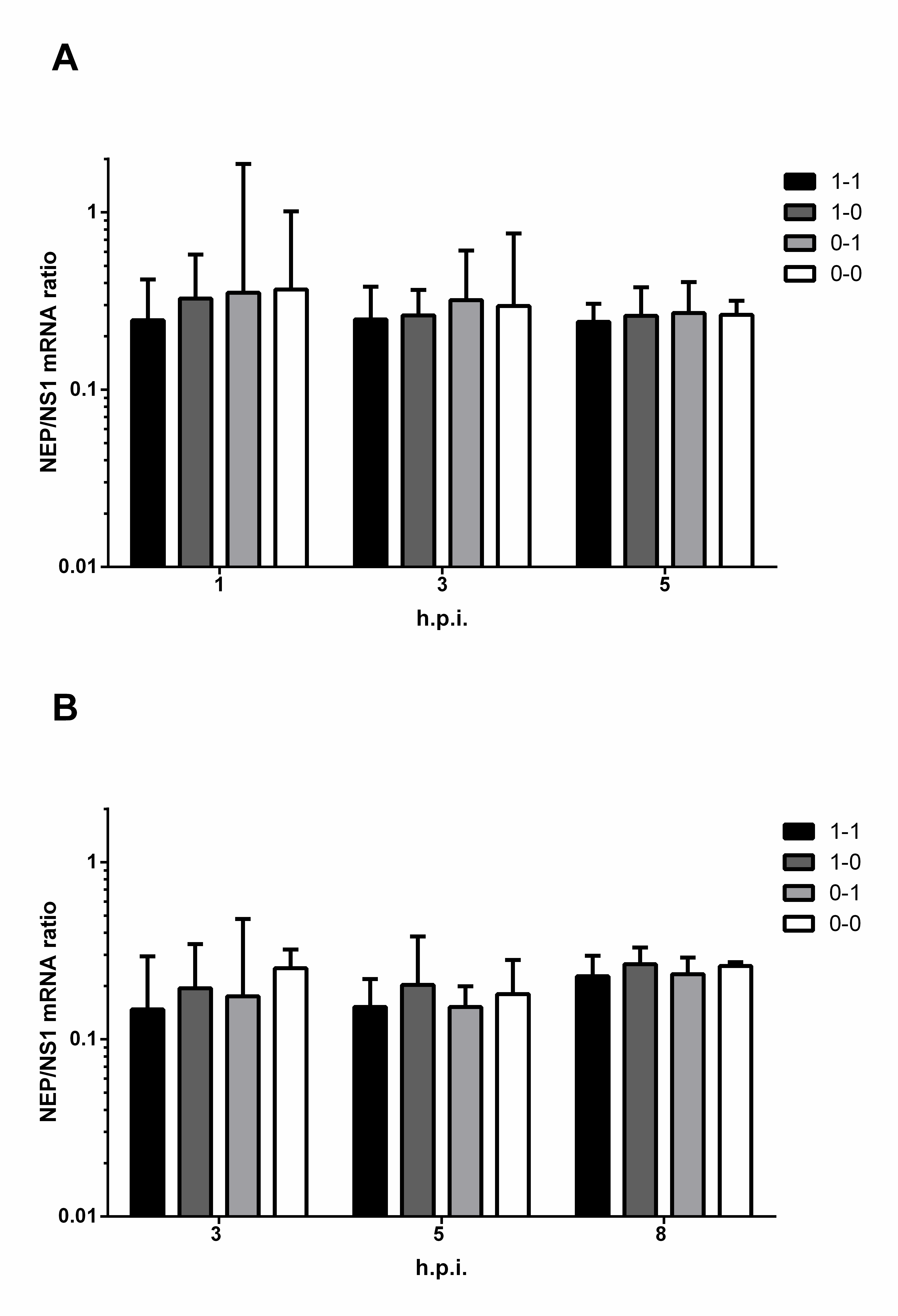
**

**Fig. S2.** Relative NEP/NS1 mRNA expression levels in cells infected by assembled viruses featuring different NS RNA secondary structures**.** MDCK (a) or A549 (b) cell cultures were infected at moi=1 TCID_50_/cell. NEP and NS1 mRNA were measured at the indicated time points. NEP mRNA was quantified by qRT-PCR using primers adapted from [Jiang et al., 2016]. Relative mRNA quantity was evaluated by the 2^−ΔCt^ method. Error bars represent geometric mean ± 95% CI.
